# Corrosion Processes on Weathering Steel Bridges Influenced by Deposition of De-Icing Salts

**DOI:** 10.3390/ma12071089

**Published:** 2019-04-02

**Authors:** Vít Křivý, Monika Kubzová, Petr Konečný, Kateřina Kreislová

**Affiliations:** 1 Department of Building Structures, Faculty of Civil Engineering, VSB-Technical University of Ostrava, L. Podeste 1875, 708 00 Ostrava, Czech Republic; vit.krivy@vsb.cz; 2 Department of Structural Mechanics, Faculty of Civil Engineering, VSB-Technical University of Ostrava, L. Podeste 1875, 708 00 Ostrava, Czech Republic; petr.konecny@vsb.cz; 3 SVUOM Ltd., U Mestanskeho Pivovaru 934/4, 170 00 Prague 7, Czech Republic; kreislova@svuom.cz

**Keywords:** deposition rate of chlorides, corrosion samples, corrosion layer, weathering steel, experimental tests, steel bridges

## Abstract

The safety and durability of bridges designed from weathering steels are conditioned by the development of a sufficiently protective layer of corrosion products. Air pollution, microclimate around the bridge, time of wetness, structural solution of the bridge, and the position and orientation of the surface within the bridge structure all influence the development of protective layers on the surface of the weathering steel. In this article, attention is focused mainly on the microclimatic effects resulting from the road traffic under the bridge. The influence of chloride deposition on the development of corrosion products is evaluated using experimental in situ testing. Two neighboring bridges made of weathering steel and crossing different types of obstacles were selected for this experiment. Relations and dependences between the measured parameters (deposition rate of chlorides, corrosion rates, thickness of corrosion products and the amount of chlorides in corrosion products) are evaluated and discussed.

## 1. Introduction

During the construction of the highway network of the Czech Republic between 2000 and 2010, a number of bridges with a weathering steel supporting structure were built. Most of these bridges are located on the D1 highway in Ostrava and its surroundings. The girder bridges are designed to be coupled with an upper concrete deck. The steel supporting elements of the bridges were made of S355J2W weathering steel [1,2]. The economic aspect was decisive for selecting the protection strategy. The selection of a weathering steel grade for bridge girders was motivated especially by the low cost of long-term maintenance of bridges compared to traditional protection strategies based on the use of conventional structural steel protected by paint systems or other coating protections.

A static assessment should consider the effects of corrosion losses of the structural elements of the bridge [3]. One of the basic input datum entering the static analysis of the structure is therefore an accurate prediction of corrosion losses for the required lifetime of the bridge [4,5]. When designing bridges on the D1 highway, corrosion losses were determined based on a general environmental classification to the degree of corrosion category C3 [6]. During extensive inspection of the bridges after 10–15 years of operation, it has been found that a sufficient protective layer of corrosion products were developed on most bridge surfaces [7]. On the bridges, however, locally bounded surfaces with corrosive unfavorable development of corrosion layer were also identified. The main cause of local defects in the development of patina was the leakage from the non-functional bridge drainage system, especially the leakage from drainage pipes of the bridge. After removing the causes of leakage and cleaning the damaged parts of the structure, new corrosion products will develop, which after several years will acquire protective patina properties.

Defects of protective corrosion layers caused by leaking from the bridge drainage system can usually be removed without the application of anti-corrosion coatings. First of all, it is necessary to remove the leakage source and to clean the surface of the structure, including non-adherent corrosion products. A new sufficiently protective corrosion layer develops over time on this prepared surface. If the cause of unfavorable corrosion development is the microclimatic environment around the bridge, then the situation is more complex. Environmental parameters around the bridge cannot usually be changed. In order to ensure the required service life of the structure, it is therefore necessary to update the corrosion prediction model, and subsequently statically evaluate the effect of accelerated corrosion effects on the long-term reliability of the structure. If the prediction of expected corrosion losses is statically unacceptable, additional corrosion protection should be applied to corrosion-damaged surfaces, for example in the form of zinc-based coatings [8] in the case of structural steel or epoxy-coating, in the case of concrete reinforcement [9]. Structural engineers are responsible for the reliable design of bridge construction and also for the design of their repairs. The accessibility of practical information related to the climatic conditions and their effect is crucial with respect to selection of the appropriate type of corrosion protection while respecting local environmental characteristics around the bridge.

**Figure 1:**
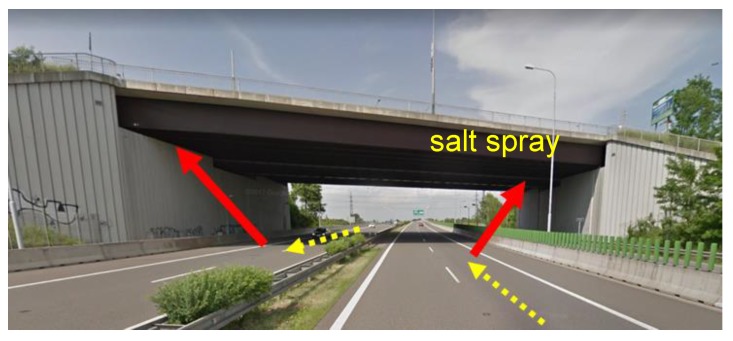
Tunnel-like conditions under the bridge (road bridge over highway D1 in the city of Ostrava, Czech Republic).

For some bridge constructions, inspections have identified surfaces that are not exposed to direct leakage, yet they do not develop a sufficiently protective layer of corrosion products [10]. In all observed cases, the upper surface of the bottom flanges of the main bridge girders was damaged. This type of corrosion damage was identified only at the bridges or their parts that are above road with intensive traffic. For other types of obstacles (vegetation, rivers and lakes, railway lines) there is no corrosion damage on the bottom flanges. It has been found that the development of unprotected corrosion layers is associated with the increased deposition of aerosol and airborne impurities on these surfaces. The development of no protective corrosion products on the bottom flanges is mainly caused by the increased deposition of chlorides that are bound to dust particles and aerosols. Chloride sources are chemical de-icing agents used in winter road maintenance. The amount of deposited chlorides is significantly affected by the structural solution of the bridge (see Figure 1).

**Figure 2:**
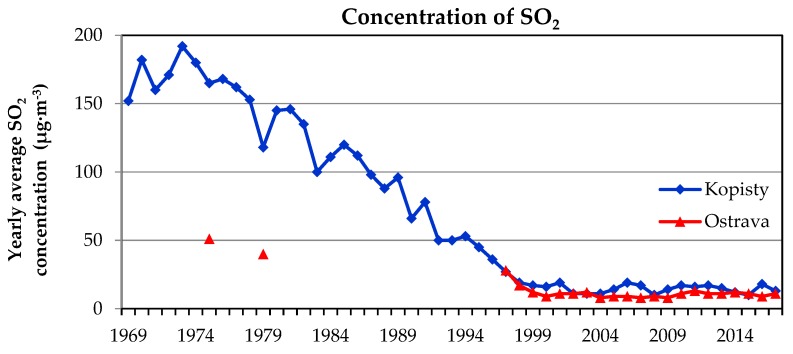
Decreasing of concentration of SO_2_ in locality Kopisty and Ostrava (Czech Republic).

Experiences with real bridge operations show that when designing a structure, it is necessary to pay more attention to the prediction of local microclimatic conditions that may be created around the planned building. The general classification of the site into a degree of corrosive category without linking to the dispositional and structural design of the proposed bridge and surrounding buildings is insufficient. The bridges on the D1 highway in Ostrava were designed based on corrosion maps, which mainly considered the concentration of SO_2_ in the atmosphere as the main corrosion factor [11]. The SO_2_ concentration in the air reached maximum in Central Europe in the 1970s [12,13]. At the time of design and construction of bridges on the D1 highway, these values were significantly lower, see Figure 2.

A low concentration of SO_2_ in the air has an impact on the development of corrosion products on the surface of weathering steel. Especially for sheltered surfaces (typically surfaces protected by the upper bridge deck), the development of corrosion products is very slow with a corrosion rate corresponding to the degree of corrosion category C1 to C2 [14]. It turns out that these surfaces with a slowly developing patina can be very negatively affected by the increased deposition of chlorides [15,16].

This article therefore pays attention to the development of corrosion products on the steel structure of the bridge protected by the upper concrete deck, which are affected by the increased deposition of chlorides. Attention is paid to the study of the microclimatic environment around the bridge and to the influence of the design and layout of the bridge on the development of corrosion products. The article further develops the findings described in previous publications of the authors, especially the reference [10]. Compared to previous research of the authors [7,10,17], the current article goes into more depth, bringing (a) a detailed analysis of the causes of the different corrosion development on the inner and outer flanges of the main girders affected by chloride deposition; (b) the practical application of visual evaluation of corrosion products; (c) analysis of the steel surface under the corrosion layer in relation to the chloride deposition rate; (d) analysis of two-year continuous measurement of chloride deposition, and (e) detailed elemental and phase analysis of corrosion products on typical surfaces of bridge. It is worth mentioning that compared to the available research, such as [18], the presented results are unique because they represent local data necessary for the design of the bridges in the Moravian-Silesian region of the Czech Republic.

## 2. Materials and Methods

### 2.1. Bridges Selected for Experimental Testing

Two bridge structures were selected for the experimental measurement of the deposition of chlorides and their effect on the development of corrosion products. Both bridges are located in Ostrava (Czech Republic):

- Bridge B1: Weathering steel bridge on the road No. 479 over highway D1 (built in 2001);
- Bridge B2: Weathering steel bridge on the road No. 479 over railway line (built in 1983).

**Figure 3:**
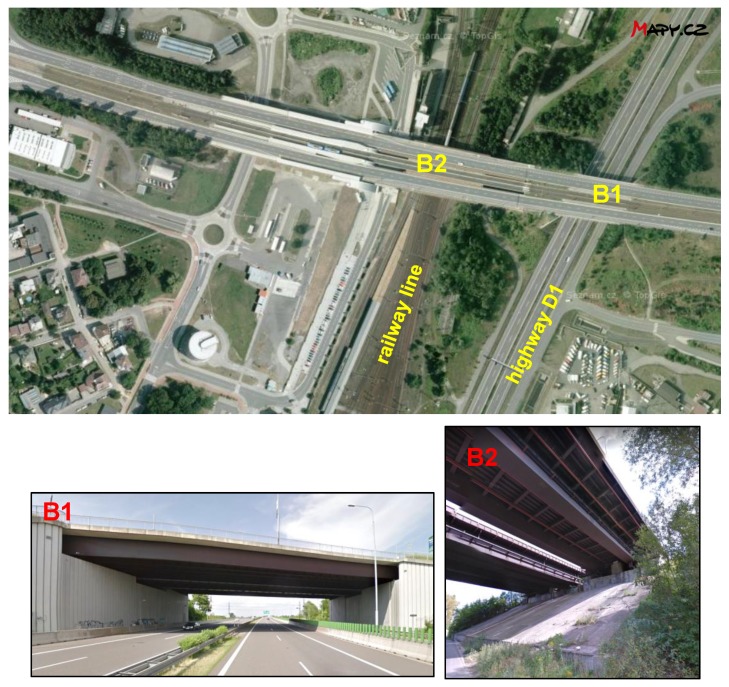
Location of the selected bridge structures.

The location of the bridges can be characterized in accordance with EN ISO 9223 [19] as an urban environment with a corrosion category for carbon steels C2 to C3. The impact of a marine environment [20] is negligible as the distance to the nearest coast is approximately 500 km. The rated bridge structures are located on the same roadway. The supporting structures of both bridges are made of S355J2W weathering steel. The distance between the centers of the bridges is approximately 200 m (see Figure 3). The bridge structures are similar in design: girder bridges with an upper deck. Both bridges are designed with sufficient overhang of the upper bridge deck over the tops of the main girders. The surfaces of the main girders of both bridges correspond to sheltered exposure conditions, except for the outer girders, which can be partially washed by wind-driven rainfall. The structural design of the B1 and B2 bridges is therefore very similar with regard to the settlement of chloride and dust particles stirred up by traffic leading across the bridge.

In terms of the development of corrosion products, the two bridges compared differ only in the nature of the bridged obstacle. Bridge B1 is located above the highway and exposed to intense road traffic beneath the bridge. The fast-moving vehicles on the D1 highway are a significant source of chlorides spreading into the environment and onto the B1 bridge’s supporting girder structure. In winter, chlorides are dispersed over the surfaces of the structure mainly as an aerosol, while in the rest of the year, they are spread as dust deposits. Bridge B1 is specific in its construction design, however, with bridge supports forming perpendicular walls directly adjacent to the sides of the motorway. The environment under the bridge is also influenced by the geometric ground plan, the bridge having a large width (41.0 m) to length (51.4 m) ratio. In addition to 4 lanes for road transport, the bridge includes two tramway tracks. The aerosol and dust deposits stirred up by automobiles traveling on the highway therefore cannot spread in the transverse direction to the motorway axis. This means there is no dispersion of particles into the surrounding environment and the surfaces of the bridge structure are exposed to increased sedimentation of airborne impurities, including chlorides [21]. Bridge B2 is located above a railway line and a local road with minimal traffic. Bridge B2 therefore has no significant source of chloride proliferation beneath it. Road traffic on both bridges is identical, and the results of the measurements could therefore be used to evaluate the effect of traffic under the bridge structure on the development of corrosion products.

### 2.2. Monitoring of Deposition Rate of Chlorides

**Figure 4:**
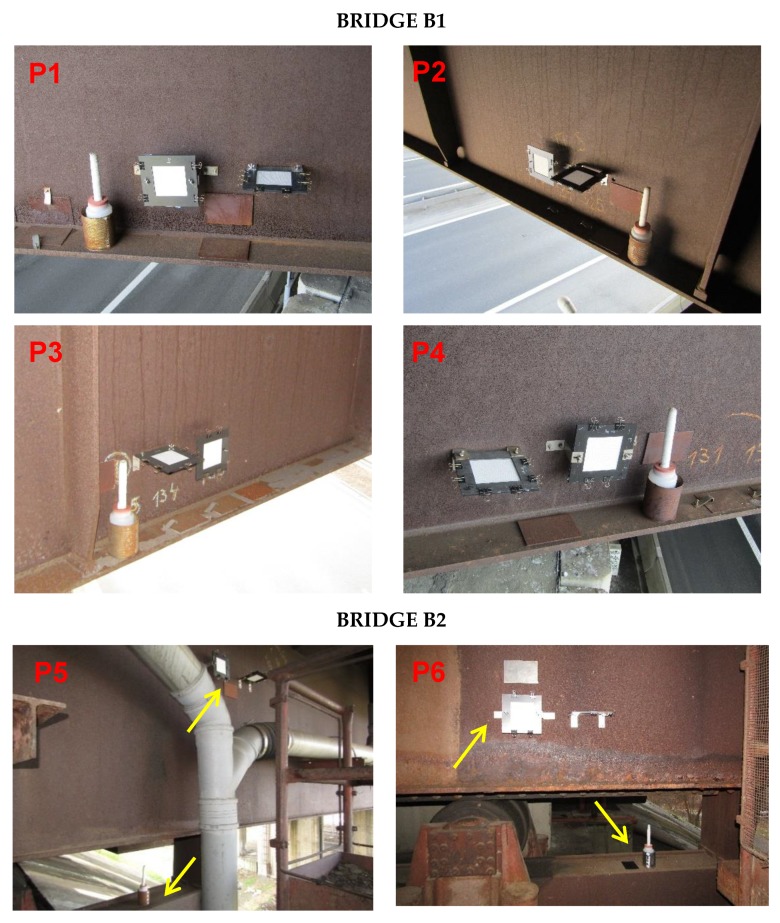
Selected positions for experimental testing on bridges B1 and B2.

Two basic methods were selected for measuring the deposition rate of chlorides (*S*_d_), in accordance with EN ISO 9225 [22]: the wet candle method and the dry plate method. Standard measuring assemblies were modified so that they could be placed at selected positions on the bridges. The principles of chloride deposition measurement using individual methods are given in [23,24]. Chloride deposition analysis using the spectrophotometric method was performed on the collected samples at regular monthly intervals. With this measurement, the deposition rates could be obtained for individual selected positions or for the total amount of chlorides deposited on the surface in the monitored period. Chloride deposition measurement assemblies were placed on bridges B1 and B2 in December 2016, and continuous measurements are still underway. Measurement of the deposition rate of chlorides was performed at the following positions (see Figure 4):

- P1: Bridge B1—external girder, north orientation (in the direction of transport under the bridge);
- P2: Bridge B1—internal girder, north orientation (in the direction of transport under the bridge);
- P3: Bridge B1—internal girder, south orientation (opposite to the direction of transport under the bridge);
- P4: Bridge B1—external girder, south orientation (opposite to the direction of transport under the bridge);
- P5: Bridge B2—external girder, north orientation;
- P6: Bridge B2—internal girder, north orientation.

### 2.3. Monitoring of Development of Corrosion Products

Corrosion coupons of S355J2WP steel (Corten A) were placed on the bridge structures to monitor the development of the corrosion layer on the selected positions. Coupons were made in standard sizes 150 × 100 × 1.5 mm^3^ in accordance with EN ISO 9226 [23]. Only the front of the corrosion coupon was exposed to the effects of the environment. The reverse side adjacent to the steel structure of the bridge was protected from environmental influences with anti-corrosion protection. Corrosion coupons were attached to the bridge structure using a set of thin magnets. Coupons in horizontal and vertical positions were placed in the same positions as chloride deposition assemblies (see Figure 4). An analysis of corrosion products was performed after one year of exposure to corrosion environment. The main monitored characteristics of corrosion products included:

- Appearance of the corrosion layer surface and surface of the steel beneath the layer of corrosion products;
- Thickness of the corrosion product layer after one year of exposure (*t*_corr_);
- Corrosion loss after one year of exposure (*r*_corr_);
- Representation of individual elements in corrosion products (primarily Cl, Si and Al);
- Representation of individual phases in the corrosion layer.

Visual evaluation of the surface of the corrosion layer was supplemented by an evaluation of the proportions of adherent and non-adherent rust layers using the Scotch-tape test. In this method, Scotch-tape is pressed against the tested surface. The tape is pressed against the surface lightly and uniformly with a rubber roller or finger. After removal, the tape is attached to a sheet, such as a transparency, and kept in a record. Making a photocopy of the sheet is useful. The frequency and size of the captured rust particles is then evaluated. Fine and uniformly distributed rust particles less than 1 mm in size are typical on fully protective patina layers. A greater portion of rust particles larger than 5 mm is typical for corrosion layers that may have limited protective ability.

The surface of the steel under the corrosion product layer was visually evaluated using the Keyence VHX-5000 3D microscope (Osaka, Japan). Using the microscope, the frequency, diameter and depth of the pits on the surface of the steel under the corrosion layer were measured.

The thickness of the corrosion products (*t*_corr_) was measured using the magnetic-induction method with a PosiTector Smartlink and F-probe (DeFelsko Corporation, Ogdensburg, NY, USA). The thickness of the corrosion products was determined as the average of the 15 measured sites on the surface of the corrosion layer. The measurement sites were selected evenly along the surface of the corrosion specimen. Corrosion losses (*r*_corr_) were determined using standard gravimetric methods in accordance with ISO 8407 [24]. The corrosion loss was determined by calculation based on the difference between the original weight and the weight after removal of the corrosion layer in an inhibited hydrochloric acid solution.

A layer of corrosion products was mechanically removed from the corrosion samples to determine the chemical composition and the representation of the individual phases in the corrosion layer. The representation of individual elements in the corrosion layer was determined using the atomic absorption spectroscopy with a Agilent 280 FS AA Spectrometer (GBC Scientific Equipment, Braeside, Australia). The phase composition of the corrosion products was determined with a Philips X’pert Pro powder diffractometer (Amsterdam, The Netherlands) in a Bragg-Brentano paraphilic geometry using Co Kα radiation (λ = 1.7903 Å, *U* = 35 kV, *I* = 40 mA). Data evaluation was performed using the HighScore Plus 4.0. program. The monitored phases of the corrosion product layer included goethite (α-FeOOH), lepidocrocite (γ-FeOOH); akaganeite (β-FeOOH) and magnetite (Fe_3_O_4_). Based on the observed phase composition of the corrosion layer, the value of the PAI_α_ and PAI_β_ indexes (protective ability index) were determined in accordance with [25,26].

## 3. Results

The following chapter presents the results of the experimentally determined characteristics of the evaluated corrosion products and the results of the deposition rates of chlorides for the measured positions P1 to P6.

### 3.1. Visual Evaluation of Corrosion Products

Visual evaluation of corrosion products was performed both for the corrosion layers developed directly on the bridge B1 (exposure period of 17 years), and for the corrosion products developed on the surface of the exposed samples (exposure period of 1 year).

**Figure 5:**
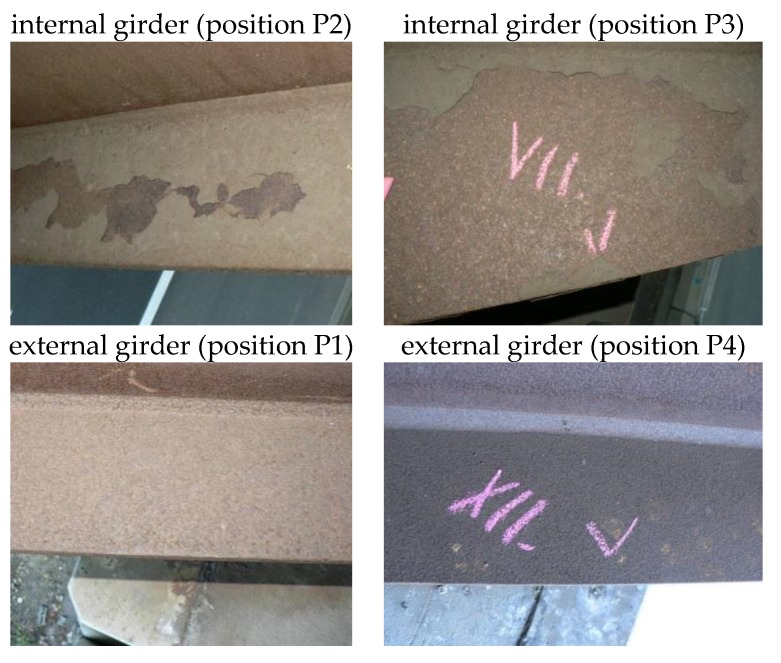
Development of corrosion products on the bottom flanges of the main girders on bridge B1: above—detrimental development on inner surfaces; below—acceptable development on outer surfaces.

A sufficiently protective layer of corrosion products, typical of sheltered exposures, has developed on bridge B1. An exception is on the upper surface of the bottom flanges of the inner girders (see Figure 5), where non-adherent layers of corrosion products have formed, with a large proportion of deposited impurities on the surface. Non-adherent corrosion products have formed on both the northern and southern sides of the inner main girders, i.e., both in the direction and opposite direction of traffic under the bridge construction. Compared to the bottom flanges of internal girders, a compact and sufficiently adherent layer of corrosion products has developed on the outer flanges of exterior main girders (see Figure 5).

**Figure 6:**
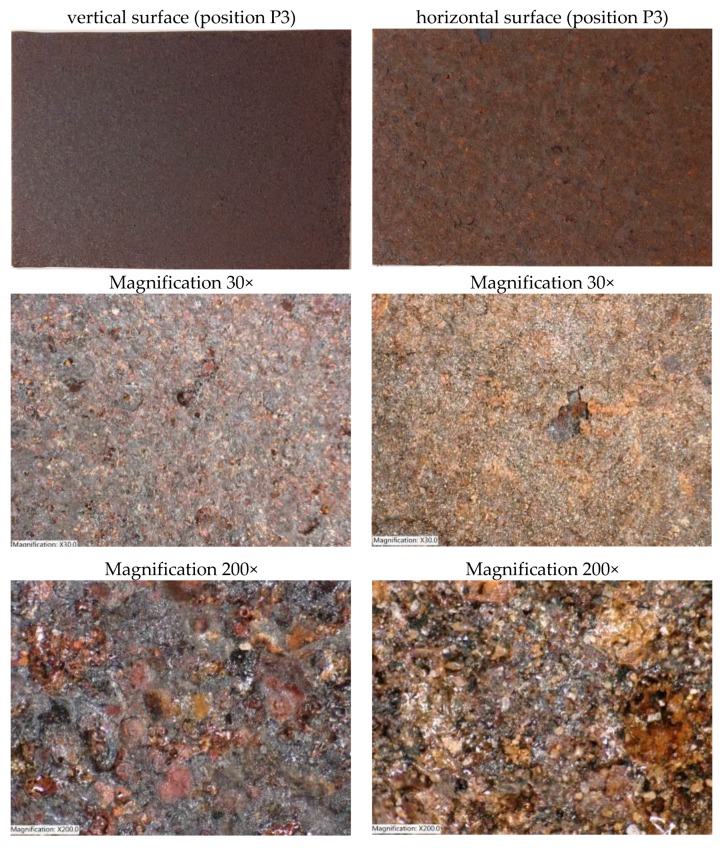
Bridge B1, position P3—layer of corrosion products on the surfaces of exposed samples after one year of exposure.

Figure 6 shows the surface of exposed steel samples in the vertical and horizontal orientation for position P3. On the horizontally-oriented sample, a larger and less compact layer of corrosion products has developed compared to the vertically-oriented sample. The corresponding corrosion layers also have a different coloration. In the horizontally-oriented sample, a higher proportion of settled pollution is evident.

**Figure 7:**
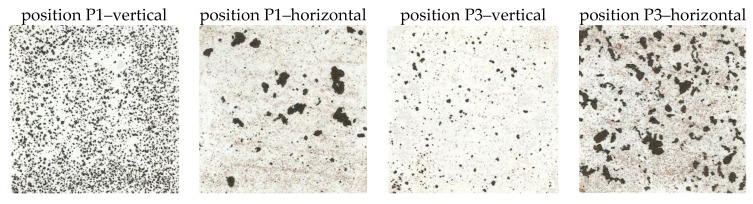
Scotch-tape test for positions P1 and P3 for vertical and horizontal placement of the surface of the steel coupon and one-year exposure.

Adhesion of corrosion products was assessed using the Scotch-tape test method. Imprints were collected after a one-year exposure of the corrosion samples (see Figure 7). Small, evenly distributed corrosion particles less than 1 mm in size were collected from both vertically-oriented surfaces. On both horizontally-oriented surfaces, larger particles with a size of approximately 5 mm were collected. Higher proportions of large corrosion particles were identified on the inner main girder (horizontal surface at position P3).

### 3.2. Steel Surface under Corrosion Layer

**Figure 8:**
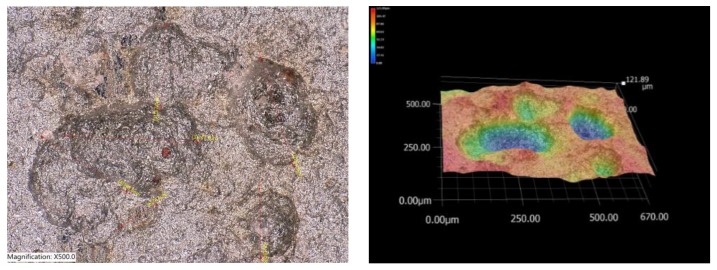
Structure of the steel surface under the corrosion layer.

The frequency and size of the individual corrosion pits were evaluated using a Keyence VHX500 3D microscope. Figure 8 shows a selected example of the surface of a corrosion sample located on the bottom flange of the inner main girder (sample from position P3).

On each evaluated surface, four positions were focused at 500× magnification. The area under review was 670 μm × 500 μm. The average number of pits in the area under observation and average values of depth and diameter of pits are shown in Table 1.

Vertically-oriented surfaces give rise to a larger number of pits which, however, have a smaller diameter and depth than on the horizontal surface. For example, in the samples exposed at positions P1, 32 corrosive pits with an average depth of 35 μm and diameter of 69 μm were found on the vertically-oriented surface after removal of the corrosion layer. On the horizontally-oriented surface at P1, 18 pits with an average depth of 59 μm and diameter of 84 μm were identified.

### 3.3. Deposition Rate of Chlorides

Chloride deposition has been monitored continuously on selected bridge structures since December 2016. Data for 24 months are available and can be separated into two periods (December 2016 to November 2017 and December 2017 to November 2018).

**Figure 9:**
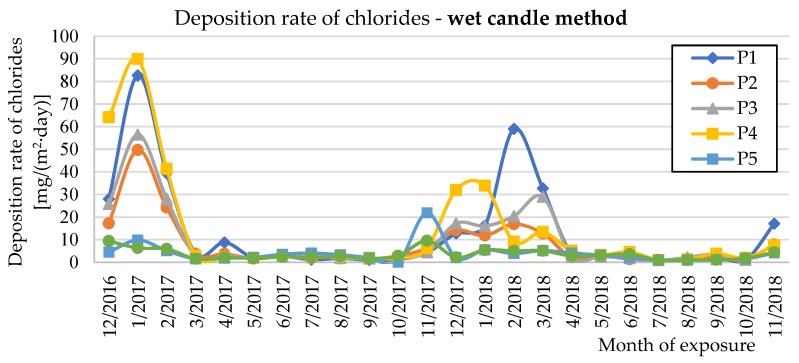
Deposition rates of chlorides determined by the wet candle method.

**Figure 10:**
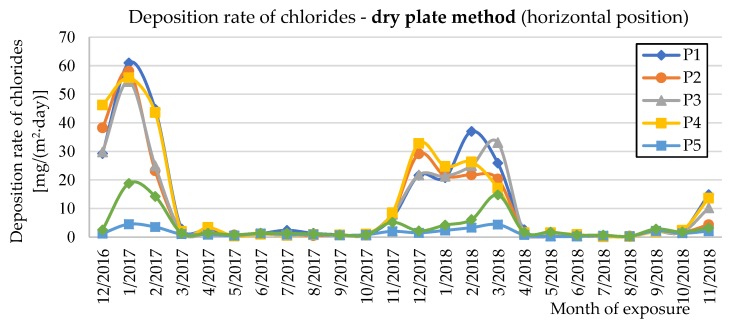
Deposition rates of chlorides determined by the dry plate method (horizontal position).

**Figure 11:**
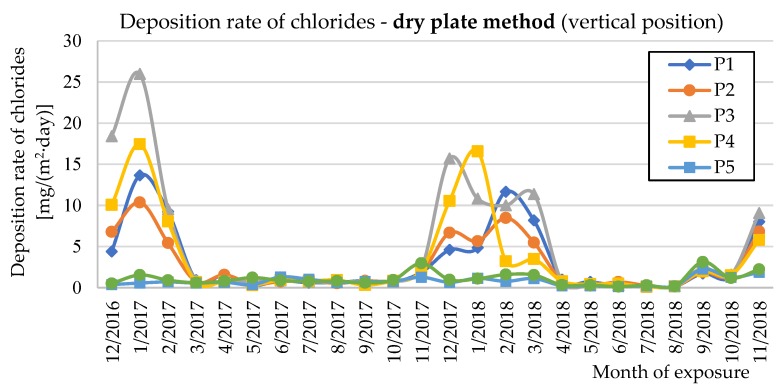
Deposition rates of chlorides determined by the dry plate method (vertical position).

The deposition rate of chlorides (*S*_d_) was measured using the wet candle method (*S*_d,c_) and the dry plate method in vertical and horizontal positions (*S*_d,p_) in accordance with EN ISO 9225 [22]. Figure 9, Figure 10 and Figure 11 show the experimentally determined deposition rates of chlorides for the individual methods and the measured surfaces of the two evaluated bridge structures. The annual average values of deposition rates of chlorides are given in Table 2 for both seasons measured and individual measurement methods.

### 3.4. Average Thickness of Corrosion Products after 1-Year Exposure

Table 3 shows the corrosion layer thickness (*t*_corr_) of steel samples exposed at selected positions between December 2016 and November 2017 and between December 2017 and November 2018 (the average of 15 measurements is shown in the table).

Position P5 was significantly affected by the failure of the bridge expansion joint. The horizontally-oriented steel coupon has been influenced by the direct flow of salt solution from the roadway. The 190.0 and 192.3 μm values therefore cannot be used to evaluate the effect of the deposition of chlorides in the form of aerosol or dust particles. Details on the development of corrosion products exposed to leaking from expansion joints are given in [7].

### 3.5. Corrosion Losses after 1-Year Exposure

Corrosion loss values for samples exposed for one year are listed in Table 4. Results for the 2016/2017 measurement period are available.

### 3.6. Content of Chlorides in Corrosion Products

The amount of chlorides deposited in the corrosion products was determined in an elemental analysis of the corrosion layer. The analysis was performed on corrosion products that had formed on the steel coupons after one year of exposure. Table 5 lists the results for the 2016/2017 measurement period. The results of the elemental analysis were converted into units of the sample surface. In addition to chlorides, the representation of aluminum and silicon are also listed in Table 5. The presence of these elements in corrosion products is mainly due to the settling of dust particles.

### 3.7. Phases in Corrosion Layer

X-ray diffraction analysis revealed the representation of individual phases in corrosion product layers. The representation of individual phases, including the specified PA indexes, is given in Table 6.

## 4. Discussion

The next chapter discusses the results of the measurements performed on two selected bridge structures in Ostrava. From the experimentally obtained data, the effect of microclimatic conditions under the bridge structure on the development of corrosion products on typical surfaces of the girder bridges can be evaluated. Bridge B1 conveys traffic over the busy D1 highway, which has chemical de-icing agents applied during the winter season. Bridge B2 conveys traffic over a railway line and is not affected by the negative impact of intense road traffic under the bridge. A comparison of the results from both bridges can therefore be used to evaluate the specific microclimatic influences of bridge structures spanning roads with high traffic volumes.

### 4.1. Influence of Orientation of the Surface

The development of corrosion products on the surface of weathering steel is different for vertically and horizontally positioned surfaces of bridge constructions (see Table 3). The thicknesses of corrosion products (*t*_corr_) observed from the one-year exposures in the periods 2016/2017 and 2017/2018 show similar dependencies with respect to surface orientation. The thickness of corrosion products on the vertical surfaces (the webs of the main girders) of both bridge structures is much smaller compared to the horizontal surfaces (bottom flanges of the main girders). On horizontally-oriented surfaces, after a one-year exposure, up to three times the thickness of the corrosion layer was observed compared to the adjacent vertically-oriented surface. Similar ratios were also found between horizontal and vertical surfaces when evaluating corrosion losses (*r*_corr_).

The differences between horizontally and vertically oriented surfaces were also observable in other characteristics. On horizontally-oriented dry plates, up to five times the deposition rate of chlorides (*S*_d,p_) was found compared to the vertically-oriented dry plates (see Table 2). In horizontally-oriented surfaces, a significantly higher proportion of chlorine (*m*_Cl_) was observed in corrosion products on the exposed samples (see Table 5). For all horizontal surfaces, the corrosion layer was found to have a strong representation of the akageneite phase, which is characteristic of surfaces exposed to chloride deposition. The increased deposition of dirt and dust was also observed on horizontal surfaces, resulting in an increased presence of aluminum and silicon in corrosion products. Different corrosion development on horizontal and vertical surfaces was also evident from visual inspections (see Section 3.1) and from the structural analysis of the steel surface beneath a removed corrosion layer (see Section 3.2). 

### 4.2. Differences between External and Internal Girders

**Figure 12:**
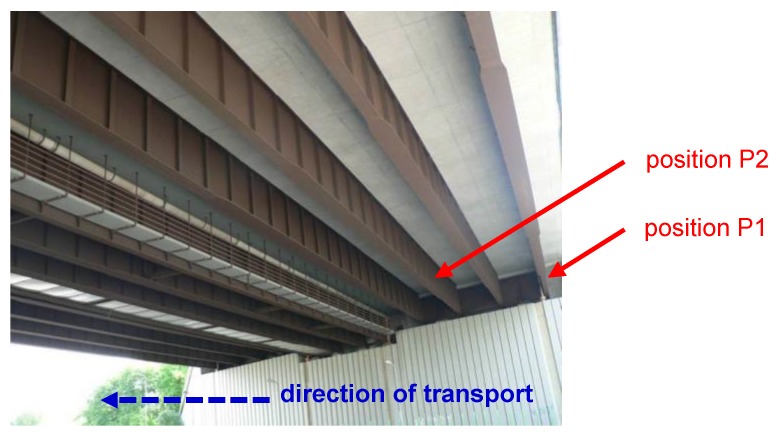
Direction of traffic and positions P1 and P2.

In evaluating corrosion products (*t*_corr_) and corrosion losses (*r*_corr_) after one year of exposure on corrosion samples, no significant differences were found between the outer and inner surfaces of the bridge (however, for horizontally-oriented samples, significantly higher values of the two monitored quantities were recorded compared to the adjacent vertical surfaces). However, the differences between the outer and inner surfaces of the bridge were reflected in the weight of chlorine (*m*_Cl_) in the corrosion products. Bridge B1 was found to have a higher weight of chlorine in the corrosion products at the P2 and P3 internal surfaces, although higher deposition rates (*S*_d,c_), determined using the standard wet-candle method, were found on the outer surfaces at P1 and P4. The findings can be demonstrated on two comparable horizontal surfaces with the same orientation in the direction of traffic under the bridge (see Figure 5 and Figure 12):

- Bridge B1, position P1 (north orientation, horizontal surface of bottom flange of external main girder), 1-year exposure (2016/2017): *m*_Cl_ = 5.68 g·m^−2^; *S*_d_ = 14.72 mg/(m^2^·day);
- Bridge B1, position P2 (north orientation, horizontal surface of bottom flange of internal main girder), 1-year exposure (2016/2017): *m*_Cl_ = 11.40 g·m^−2^; *S*_d_ = 9.80 mg/(m^2^·day).

The apparent disproportion between the amount of chlorides found in the corrosion products (*m*_Cl_) and the deposition rates of chlorides (*S*_d_) can be explained by the fact that although the external surfaces of the bridge are more exposed to chlorides and impurities, the external surfaces are also regularly cleaned by wind and rainfall. The amount of chlorine found in the corrosion products of the external surfaces of bridge B1 was therefore less than the internal surfaces. This phenomenon negatively manifests in long-term development, where non-adherent layers of corrosion products with a large proportion of settled impurities form on the upper surfaces of the bottom flanges of the inner main girders (see inner surfaces shown in Figure 5).

From the above results, it follows that in predicting the long-term development of corrosion products based on year-long testing, it is necessary to analyze the sum of all information obtained from the corrosion tests. The results of corrosion thickness (*t*_corr_) and corrosion loss (*r*_corr_) measurements after one year of exposure may not provide sufficiently accurate data about the future development of corrosion products. It is therefore very important to supplement the assessment of the development of corrosion products with other parameters: mainly visual assessment of the development of corrosion products, including evaluation of collected imprints (Scotch-tape test), and the results of chemical analysis of the corrosion layer, focusing on the elemental content that indicates increased deposition of chloride ions from road traffic. For example, corrosion loss values (*r*_corr_) measured on horizontal surfaces of the B1 bridge correspond to corrosion category C3 according to EN ISO 9223 [19]. Classification in corrosion category C3 itself, expected for an urban industrial environment based on annual corrosion tests, does not indicate the potential risk of adverse corrosive product development on the inner flanges of the bridge. Differences in corrosion loss values between internal and external surfaces will be more significant only after long-term exposure, when the structure has been exposed to increased chloride deposition [10].

### 4.3. Influence of Microclimatic Conditions under the Bridge Structure

Bridge B1 conveys traffic over the busy D1 highway, which has chemical de-icing agents applied during the winter season. Bridge B2 conveys traffic over the railway line and is not affected by the negative impact of intense road traffic under the bridge. Differences in the character of the spanned obstacle were reflected in all experimental data related to the development of corrosion products.

Figure 9, Figure 10 and Figure 11 show the deposition rates of chlorides (*S*_d_) for individual surfaces and measurement methods. The graphs show elevated deposition rates of chlorides occurring in the winter periods, when the roads are treated with chemical de-icing agents. Both observed winter periods show differences in the observed values. During winter 2016/2017, higher deposition values of chlorides were measured than in winter 2017/2018. These differences are mainly related to the current climatic conditions during the winter period. Data from two selected winter months are shown in Table 7 for clarity.

For bridge construction B2, the deposition rates of chlorides in winter periods were found to be several times lower than on the bridge B1 (for example, in January 2017, approximately nine times lower compared to the outer surfaces of the bridge B1). A comparison of the data obtained on bridges B1 and B2 shows a significant influence in the source of chlorides. The upper bridge deck with sufficient lateral overhang protects the main girders of both B1 and B2 bridges from the increased deposition of particles stirred up by traffic over the bridge. However, bridge B1 is located above a busy highway, and the construction of the bridge also creates tunnel-like conditions. The main source of elevated chloride deposition is road transport under the bridge structure.

The influence of the environment under the bridge structure is also evident from the results of the elemental analysis of the exposed samples. On the horizontal surface of the B1 bridge spanning the motorway, more than six times the difference in the presence of chlorides in corrosion products was found after one year of exposure than on a comparable surface of the bridge B2 over the railway line. Large differences are also apparent in the representation of aluminum and silicon, i.e., elements indicating increased deposition of dust particles and other impurities.

- Bridge B1, position P2 (north orientation, horizontal surface), 1-year exposure (2016/2017): *m*_Cl_ = 11.40 g·m^−2^; *m*_Al_ = 3.25 g·m^−2^; *m*_Si_ = 16.33 g·m^−2^;
- Bridge B2, position P6 (north orientation, horizontal surface), 1-year exposure (2016/2017): *m*_Cl_ = 1.69 g·m^−2^; *m*_Al_ = 0.49 g·m^−2^; *m*_Si_ = 2.03 g·m^−2^.

On the horizontal structures of bridge B1, the phase analysis revealed a very strong presence of akaganeite, which is produced in an environment with a high occurrence of chlorides. On bridge structure B2, lepidocrocite was predominantly represented on horizontal surfaces in the corrosion layer. Although using the values of PA indices set on the developing layer of corrosion products to estimate the protective effects of fully-developed patina is not suitable, it can be inferred by comparing the PAI_β_ to influence corrosion processes due to chlorides. The values of the PAI_β_ indexes were higher for bridge B1 than for bridge B2 (see Table 6).

The different environments beneath both tested bridges also manifested in the values of corrosion thickness (*t*_corr_) and corrosion losses (*r*_corr_) determined after one year of exposure of the corrosion samples. A more than double difference was found for both monitored variables:

- Bridge B1, position P2 (north orientation, horizontal surface), 1-year exposure (2016/2017): *t*_corr_ = 188.0 µm; *r*_corr_ = 45.8 µm;
- Bridge B2, position P6 (north orientation, horizontal surface), 1-year exposure (2016/2017): *t*_corr_ = 83.0 µm; *r*_corr_ = 20.4 µm.

**Figure 13:**
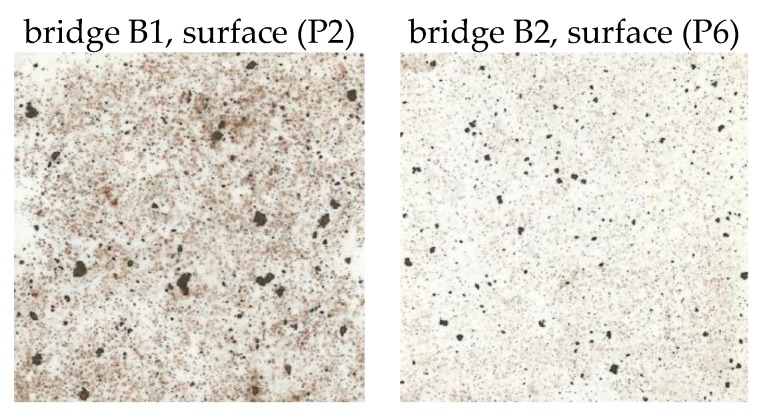
Scotch-tape tests on selected horizontal surfaces.

The different development of corrosion products on the horizontal surfaces of both evaluated bridges can also be documented based on a visual evaluation. On the bridge B1 samples, bulkier and less adherent corrosion products developed with a higher proportion of settled impurities. Visual evaluation was supplemented by an evaluation of adherent and non-adherent rust layers using the Scotch-tape test. Imprints of horizontal surfaces from the bridge B1 show an uneven distribution of trapped particles of various sizes, whereas the size of the largest particles collected on the imprint were approximately 5 mm. For bridge B2, the collected particles were evenly distributed and the particle size was up to 2 mm. Imprints from the compared surfaces at positions P2 and P6 are shown in Figure 13.

### 4.4. Brief Static Evaluation of Corrosion Weakening

The evaluated bridge structures are made of structural steel S355J2W. The main corrosion risk associated with the use of this steel grade is the corrosion weakening of the structure or its parts. Stress corrosion cracking is very unlikely. During the inspection of the bridge over the D1 highway, the real thicknesses of the bottom flanges of the main girders were checked. The measurement was performed using the ultrasonic thickness gauge. Unfortunately, the original thicknesses of structural elements from the time of the bridge construction are not available. Real corrosion losses after 17 years of bridge operation are therefore unknown. However, the actual thicknesses are still within the range of rolling tolerances of the used sheets. Therefore, it is not necessary to carry out the reinforcement of structural elements with corrosion-negative development.

From the static point of view, the greatest risk is the corrosion weakening of structural elements with low thickness: such typical components are fillet welds connecting webs and flanges of the main girders. For example, a corrosion weakening of 1 mm for a 7 mm thick filled weld can be very significant in terms of reducing the load-bearing capacity of the steel bridge structure. On the other hand, the same corrosion weakening of a 50 mm thick bottom flange does not significantly affect the load-bearing capacity of the bridge. A greater risk of failure of thin structural elements should be taken into account using suitable corrosion allowance already when designing the load-bearing structure. Corrosion allowances can be calculated in accordance with the recommendations given in [17].

Regarding the local corrosion damage, it is important to evaluate its position within the bridge structure. For example, the corrosion weakening of the main girder in the area of outer hinged supports is not usually significant in terms of the bending resistance of the bridge, as there are no bending moments acting at the end of the main girders. On the other hand, the maximum shear forces act above the supports and therefore the fillet welds and the web of the main girders are significantly stressed. In the opinion of the authors of this article, it is therefore very important that the assessment of the detected corrosion failures is carried out by a structural engineer who is familiar with the static behavior of the bridge.

## 5. Conclusions

The course of corrosion processes on bridges constructed with weathering steel is significantly influenced by the chosen structural solution of the bridge and the specifics of local microclimatic conditions. The results of the measurements performed show that the general classification of a locality according to the category of corrosion aggressiveness is not sufficiently indicative for the prediction of corrosion processes if the geometric parameters of the construction design and possible microclimatic influences are not taken into consideration.

The course of corrosion processes can be very different for the different bridge structure surfaces under consideration. This fact should be reflected in designing structures [17] as well as in regular maintenance [27]. Vertically-oriented surfaces (typically webs of main girders) usually develop a uniform, thin corrosion layer typical of sheltered exposures. The patina on soffit surfaces also has similar parameters. The most complex issue is predicting the development of corrosion products on horizontal surfaces, which can be affected by increased settling of airborne particles. If the principles of a suitable design are observed and the bridge is placed in suitable microclimatic conditions, a sufficiently protective patina develops on the horizontal surfaces. By contrast, there is a risk of creating non-protective corrosion products associated with a technically unacceptable corrosive weakening of the structure.

One of the decisive factors influencing the local microclimate in the vicinity of a bridge is the effect of chlorides, which are deposited on bridge structure elements mainly due to traffic on adjacent roads. De-icing salts used for winter road maintenance are a source of chlorides. Some significant findings have been determined from the results of measuring chloride deposition. Measurements show that in the case of bridges with suitable structural arrangements, it is possible to significantly eliminate the amount of deposited chlorides, the source of which is traffic over the bridge structure. Increased chloride deposition, however, may occur due to intense road traffic under the bridge structure. At present, not enough experimentally validated data is available to define the specific dimensional and structural requirements, which if applied, decreases chloride deposition from traffic under the bridge to a level that does not limit the beneficial development of patina on bridge structure components. Therefore, it is necessary to at least observe the basic rules: to design structures that allow the dispersal of pollutants and aerosols into the surrounding environment and, importantly, to implement basic maintenance consisting of regular cleaning of the steel structure after the end of winter.

## Figures and Tables

**Table 1:** Pitting in steel surface after removal of corrosion products.

Bridge	Tested Position	Orientation	Number of Pits	Average Depth of Pits [µm]	Average Diameter of Pits [µm]
B1	P1	vertical	32	35	69
		horizontal	18	59	84
	P2	vertical	24	37	67
		horizontal	19	47	82
	P3	vertical	16	29	77
		horizontal	16	56	94
	P4	vertical	26	31	65
		horizontal	7	63	132
B2	P5	vertical	36	22	51
		horizontal	19	41	73
	P6	vertical	33	24	56
		horizontal	19	40	89

**Table 2:** Average annual deposition rates of chlorides for the two measured seasons and individual methods of measurement [mg/(m^2^·day)].

Bridge	Tested Position	Orientation	*S*_d,c_ (2016/2017) [mg/(m^2^·day)]	*S*_d,p_ (2016/2017) [mg/(m^2^·day)]	*S*_d,c_ (2017/2018) [mg/(m^2^·day)]	*S*_d,p_ (2017/2018) [mg/(m^2^·day)]
B1	P1	vertical	14.72	2.97	12.63	3.56
		horizontal		12.90		10.79
	P2	vertical	9.80	2.58	6.21	3.22
		horizontal		11.38		8.66
	P3	vertical	11.13	5.20	8.73	4.43
		horizontal		10.43		7.78
	P4	vertical	18.43	3.60	9.71	3.76
		horizontal		13.69		10.28
B2	P5	vertical	5.16	0.75	2.85	0.84
		horizontal		1.53		1.61
	P6	vertical	4.03	1.05	2.99	1.08
		horizontal		4.14		3.36

**Table 3:** Average thickness of corrosion products *t*_corr_ after 1-year exposure [µm].

Bridge	Tested Position	Orientation	*t*_corr_ [µm] (2016/2017)	*t*_corr_ [µm] (2017/2018)
B1	P1	vertical	72.0	62.5
		horizontal	206.0	145.8
	P2	vertical	72.0	64.3
		horizontal	188.0	181.6
	P3	vertical	74.0	69.6
		horizontal	222.0	153.3
	P4	vertical	59.0	33.6
		horizontal	187.0	113.8
B2	P5	vertical	56.0	25.7
		horizontal	190.0 ^1^	192.3 ^1^
	P6	vertical	57.0	27.2
		horizontal	83.0	71.1

^1^ Measurement is influenced by leaking from expansion joint.

**Table 4:** Corrosion losses after 1-year exposure *r*_corr_.

Bridge	Tested Position	Orientation	*r*_corr_ [µm·year^−1^] (2016/2017)
B1	P1	vertical	19.3
		horizontal	49.2
	P2	vertical	19.0
		horizontal	45.8
	P3	vertical	22.0
		horizontal	49.4
	P4	vertical	16.1
		horizontal	49.9
B2	P5	vertical	12.6
		horizontal	46.7 ^1^
	P6	vertical	13.6
		horizontal	20.4

^1^ Measurement is influenced by leaking from expansion joint.

**Table 5:** Content of individual elements in corrosion products [g·m^−2^·year^−1^].

Bridge	Tested Position	Orientation	*m*_Cl_ (2016/2017) [g/(m^2^·year)]	*m*_Al_ (2016/2017) [g/(m^2^·year)]	*m*_Si_ (2016/2017) [g/(m^2^·year)]
B1	P1	vertical	0.66	0.33	0.17
		horizontal	5.68	0.76	3.96
	P2	vertical	0.98	0.09	0.54
		horizontal	11.40	3.25	16.33
	P3	vertical	3.18	0.16	0.82
		horizontal	11.27	0.93	4.65
	P4	vertical	0.82	0.08	0.58
		horizontal	9.53	1.04	4.73
B2	P5	vertical	0.69	0.02	0.29
		horizontal	5.41 ^1^	1.87 ^1^	8.30 ^1^
	P6	vertical	1.17	0.06	0.41
		horizontal	1.69	0.49	2.03

^1^ Measurement is influenced by leaking from expansion joint.

**Table 6:** Phases analysis of corrosion layer.

Bridge	Tested Position	Orientation	Phases	PAI_α_	PAI_β_
B1	P1	vertical	very strongly lepidocrocite; strongly akaganeite; weakly goethite	0.06	0.73
		horizontal	very strongly akaganeite; strongly goethite; weakly lepidocrocite	0.12	0.84
	P2	vertical	very strongly lepidocrocite; strongly akaganeite; weakly goethite	0.07	0.59
		horizontal	very strongly akaganeite; strongly goethite and lepidocrocite	0.06	0.94
	P3	vertical	very strongly akaganeite; weakly goethite and lepidocrocite	0.13	0.89
		horizontal	very strongly akaganeite; strongly goethite and lepidocrocite	0.09	0.96
	P4	vertical	very strongly lepidocrocite; strongly akaganeite; very weakly goethite	0.04	0.71
		horizontal	very strongly akaganeite; strongly goethite; weakly lepidocrocite	0.07	0.78
B2	P5	vertical	very strongly lepidocrocite; weakly goethite; weakly lepidocrocite	0.15	0.47
		horizontal	very strongly lepidocrocite; strongly akaganeite; weakly goethite	0.09	0.74 ^1^
	P6	vertical	very strongly lepidocrocite; strongly akaganeite; weakly goethite	0.09	0.53
		horizontal	very strongly lepidocrocite; strongly akaganeite; weakly goethite	0.03	0.63

^1^ Measurement is influenced by leaking from expansion joint.

**Table 7:** Deposition rate of chlorides (*S*_d,c_) measured by wet candle method [mg·m^−2^·day^−1^].

Bridge	Tested Position	01/2017	02/2017
B1	P1—external girder, north orientation	82.64	39.33
	P2—internal girder, north orientation	49.67	24.20
	P3—internal girder, south orientation	56.43	28.13
	P4—external girder, south orientation	89.92	41.25
B2	P5—external girder, north orientation	9.80	5.08
	P6—internal girder, north orientation	6.33	5.89
